# Chronic kidney disease in Mayer-Rokitansky-Kuster-Hauser Syndrome

**DOI:** 10.4103/0971-4065.73447

**Published:** 2010-10

**Authors:** M. M. Wani, S. A. Mir

**Affiliations:** Department of Medicine, Al-Amiri Hospital and Al Nafisi dialysis centre, Kuwait,

**Keywords:** Mullerian agenesis, amenorrhea, congenital anomalies, chronic kidney disease

## Abstract

Mayer-Rokitansky-Kuster-Hauser (MRKH) syndrome is characterized by either absence or abnormalities of the mullerian structures. It is a rare disorder, resulting in complete or partial agenesis of the uterus and cervix and primary amenorrhea. It may rarely be associated with anomalies of the urinary tract, ovaries and skeleton. Renal failure secondary to chronic tubulo-interstitial disease has been reported. We report a case of MRKH syndrome presenting late with chronic kidney disease.

## Introduction

Mayer-Rokitansky-Kuster-Hauser (MRKH) syndrome is a rare disorder characterized by the congenital absence of the uterus and upper part (2/3) of the vagina.[1,2] The prevalence has been reported as one case in 4000-5000 female births.[3] The defect involves mesodermal development and the mesonephric kidney, resulting in abnormalities in the paramesonephros (uterus and vagina) and the metanephric kidney.[1] The diagnosis can be delayed due to late presentation because of social taboos present in some conservative societies, lack of awareness among healthcare providers and atypical organ involvement. Two subtypes of MRKH have been described, only one of which is associated with renal defects.[4] A solitary kidney, either at normal location or in the pelvis, is the most frequent renal anomaly. Patients are at risk for chronic pyelonephritis, which may lead to loss of renal tissue.

## Case Report

A 23-year-old female was referred from the Urology clinic as a case of solitary pelvic kidney and azotemia. She had history of primary amenorrhea, recurrent urinary tract infections and recent onset hypertension. Examination revealed her to be of average built (body weight of 62 kg and height of 168cms), euvolemic, with no pallor, BP- 110/70 mm of Hg (on medication), and no gross skeletal abnormality or hearing defects. Her cardiovascular, abdominal and respiratory examination was normal. She had feminine body features. Her gynecological examination showed a small vaginal pouch of about 2.5 cm ending blindly. There was no family history of any gynecological or renal abnormalities.

**Figure 1:**
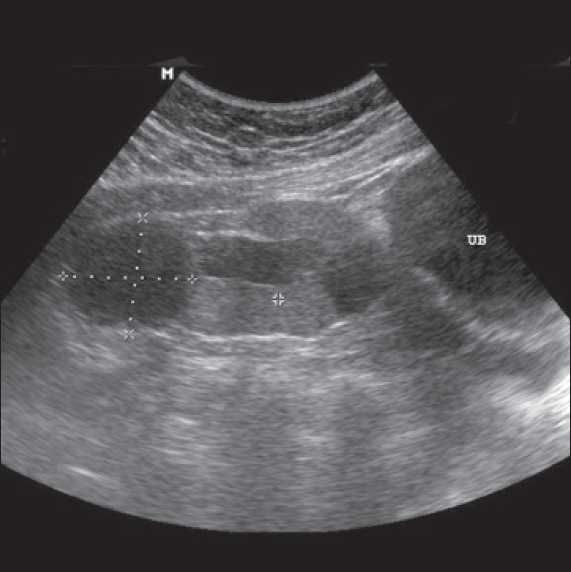
Ultrasound showing right Pelvic Kidney

**Figure 2:**
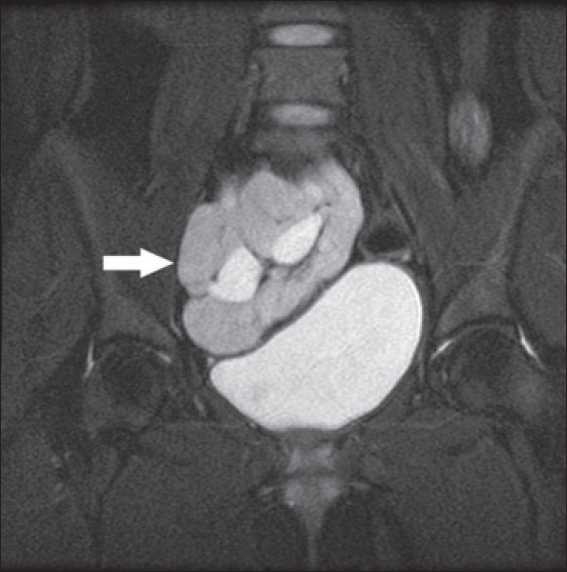
MRI showing pelvic kidney in relation to bladder

**Figure 3:**
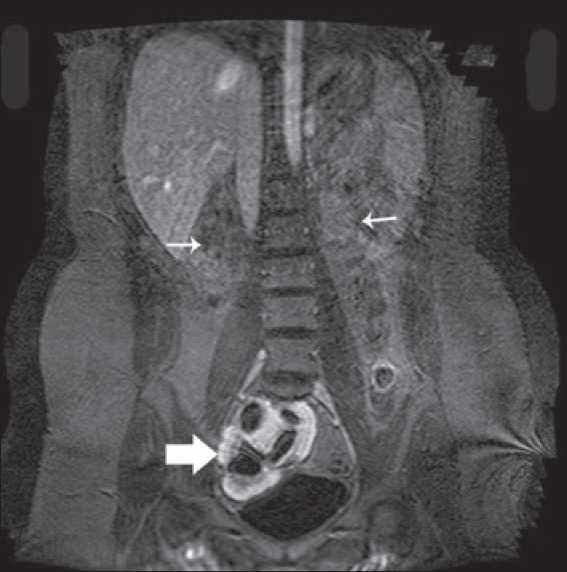
MRI showing empty renal fossae (thin arrow) and pelvic kidney in relation to bladder (thick arrow)

**Figure 4:**
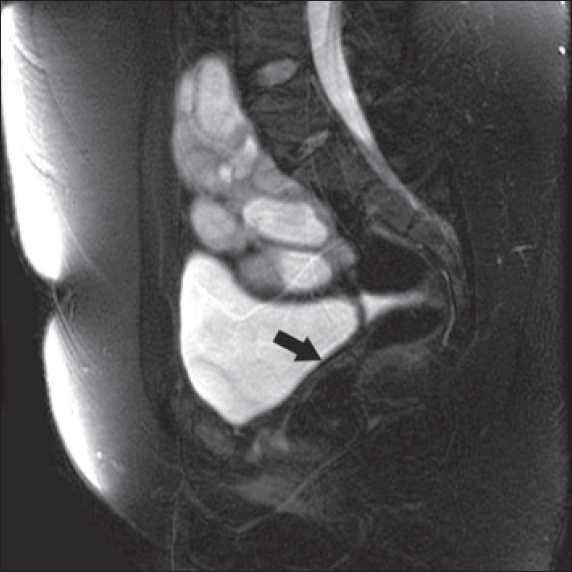
MRI showing pelvic kidney and absent uterus (Arrow)

**Figure 5:**
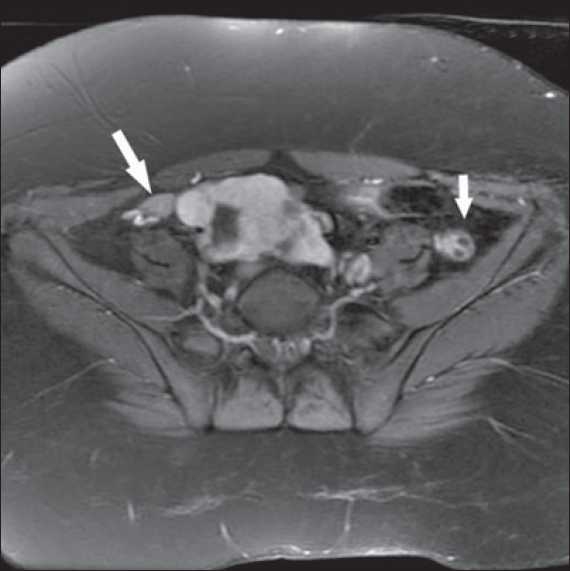
MRI showing bilateral ovaries

Investigations showed hemoglobin 117g/dL, WBC count- 7.5×10^3^/dmm^3^, platelets - 3.55×10^9^/mm^3^, urea-4.3 mmol/L, Creatinine 2mg/dl, albumin - 3.5g/dL, Calcium 9mg/dl, phosphorus- 6.5 mg/dL, sodium - 140 mmol/L, potassium- 4.8 mmol/L, PTH- 29 pmol/L. Urine examination showed 2+ proteins, leucocytes 4-6/HPF, no RBCs, 24-hour urinary protein of 1.2 gram and a creatinine clearance of 38ml/min (24-hour urinary creatinine estimation). Her hormonal profile was within normal limits. Detailed ultrasound revealed left kidney not localized, a 9.5 cm right pelvic kidney, with increased echotexture, loss of cortico-medullary differentiation, few cortical cysts and mild hydronephrosis Uterus was not seen but both ovaries were present [Figure 1]. A Magnetic Resonance Imaging (MRI) scan revealed solitary pelvic kidney in relation to right internal iliac vessels and gynecological abnormalities were confirmed [Figures 2–5]. Tc 99 DMSA scan showed a poorly outlined right pelvic kidney showing nonhomogenous tracer uptake by functional parenchymal tissue. Left kidney was not visualized. A cystoscopy done by urologists prior to her referral had shown only right ureteric orifice and absent left ureteric orifice. Genetic study revealed 46 XX karyotype.

The features are consistent with type 2 MRKH syndrome with chronic kidney disease (CKD)-stage 3. Her CKD is likely because of chronic pyelonephritis secondary to anomalies of her urinary tract. She was counseled and is on regular follow-up in renal and gynecological clinics. Because of the social stigma she refused to be assessed by a plastic surgeon as was advised by her gynecologist. She is on conservative measures for her CKD but her follow-up in renal clinic is erratic.

## Discussion

MRKH is a rare disease, diagnosed usually in a girl at the time of puberty with primary amenorrhea and normal secondary sex characteristics. Patients with MRKH syndrome have a 46 XX karyotype. The external genitalia appear normal, but only a shallow vaginal pouch is present. Ovarian function is normal.[1–3] Typical (type 1) and atypical (type 2) forms are known, symmetric muscular buds and fallopian tubes are diagnostic of type I (44% of the cases) while asymmetric muscular buds or abnormally developed fallopian tubes are diagnostic of type 2 (56% of the cases).[4–6] Anomalies of the urinary tract, ovaries, skeleton (malformations of the cervical vertebra, scoliosis of the thoracic and lumbar spine) and congenital hearing loss may occur in women with type 2 MRKH syndromes. In a 25-patient series reported,[3] the frequency of anomalies mentioned included scoliosis (20%), unilateral renal agenesis (28%), non-vertebral skeletal anomalies (16%), cardiac anomalies (16%).Differential diagnosis includes congenital absence of uterus and vagina (aplasia or agenesis), isolated vaginal atresia and androgen insensitivity.

For a long time the syndrome has been considered a sporadic anomaly, but increasing number of familial cases now support the hypothesis of a genetic cause. In the familial cases the syndrome appears to be transmitted as an autosomal dominant trait with incomplete penetrance and variable expressivity, suggesting either mutations in major developmental gene or a limited chromosomal imbalance.[7] However, the etiology of MRKH syndrome still remains unclear.

Radiological studies with laparoscopic findings were necessary previously to diagnose and classify this syndrome.[5] MRI being less invasive and expensive is now the preferred modality of defining the anatomical characteristics of this syndrome.[6]

This patient came to us with recurrent urinary tract infections, hypertension and azotemia. Her abdominal and pelvic sonography as routine screening had shown the absence of uterus, left renal agenesis and a contra-lateral pelvic kidney. These findings were confirmed by urography and MRI scan of the abdomen. Gynecologic examination showed a small vaginal pouch (2.5 cm).Thus the diagnosis of type 2 MRKH syndrome with associated congenital anomalies of the upper urinary tract was made for the first time in this lady at the age of 23 years. The frequency of renal abnormalities is reported to be about 30–40%, including unilateral agenesis, ectopia of one or both kidneys, renal hypoplasia, horse shoe kidney and hydronephrosis.[6,8] The frequency of MRKH syndrome amongst women with missing kidney is unknown. However, in 40–50% of patients with renal agenesis, an associated genital anomaly is found.[8,9] Patients with major anatomic abnormalities are at risk for chronic pyelonephritis, induced by recurrent renal infections and the scarring of chronic pyelonephritis leads to loss of renal tissue and function resulting in progression to chronic kidney disease.[4] Because of the social taboos prevalent in the Kuwaiti community this female was reluctant to come up with the full history and hence a delay in her diagnosis till 23 years of age.

In conclusion, this case report highlights the importance of need for early investigations of anatomical urogenital defects in a case of primary amenorrhea.
